# Dads in Distress: symptoms of depression and traumatic stress in fathers following poor fetal, neonatal, and maternal outcomes

**DOI:** 10.1186/s12884-022-05288-5

**Published:** 2022-12-22

**Authors:** A. Kothari, G. Bruxner, J. M. Dulhunty, E. Ballard, L. Callaway

**Affiliations:** 1 Redcliffe Hospital, Anzac Avenue, Redcliffe, Queensland 4020 Australia; 2 The University of Queensland, Brisbane, Queensland Australia; 3 QIMR Berghofer Medical Research Institute, Brisbane, Queensland Australia; 4 The Royal Brisbane and Women’s Hospital, Brisbane, Queensland Australia

**Keywords:** Paternal perinatal depression, Post-traumatic stress disorder, Post-traumatic stress symptoms, Psychiatric status rating scales

## Abstract

**Background:**

This study aims to explore the prevalence of symptoms of depression and traumatic stress in fathers in the setting of poor fetal, neonatal, and maternal outcomes.

**Methods:**

A prospective mixed-methods study was conducted at an outer metropolitan public teaching hospital in Brisbane, Australia, with quantitative results presented here. Subjects included 28 fathers whose male partners had experienced pregnancy or childbirth complicated by a significant congenital abnormality or aneuploidy, termination of pregnancy, fetal death in-utero, stillbirth, admission to the neonatal intensive care unit or special care nursery or significant maternal morbidity, such as a postpartum haemorrhage or an emergency postpartum hysterectomy. These experiences were classified into two groups: anticipatory (time to prepare) and sudden (no warning). The fathers were screened using the Edinburgh Postnatal Depression Scale (EPDS) and the Impact of Events Scale-Revised (IES-R) to assess subjective distress at 2-3 weeks (timepoint 1) and 3-4 months (timepoint 2) after the event.

**Results:**

Data for both the EPDS and IES-R scales was available for 26 fathers (92.9%) at timepoint 1 and for 15 fathers (53.6%) at timepoint 2. High overall EPDS scores (≥10) were noted in 16/27 (59.3%) fathers at timepoint 1 and 6/15 fathers (40.0%) at timepoint 2. High overall IES-R scores ≥33 were noted in 12/26 (46.2%) fathers at timepoint 1 and 4/15 fathers (26.7%) at timepoint 2. A higher percentage of fathers who experienced anticipatory events had EPDS and IES-R score above these cut-offs at timepoint 1 (8/13 or 61.5%) compared to those experiencing sudden events (8/14 or 57.1%), however, percentages were similar between groups at time point 2 (2/7 or 28.6%% and 4/8 or 50.0%, respectively). More fathers who experienced anticipatory events had IES-R scores ≥33 at timepoint 1 (7/13 or 53.8%) compared to those experiencing sudden events (5/14 or 38.0%).

**Conclusion:**

Our study indicates high rates of distress in fathers exposed to poor fetal, neonatal, and maternal outcomes, which can persist for months after the event. Increased support for fathers in this setting may be required to prevent poor mental health. Further research on the long-term effects of these adverse events is warranted.

**Supplementary Information:**

The online version contains supplementary material available at 10.1186/s12884-022-05288-5.

## Introduction

The cultural expectation for fathers to be involved in the pregnancy and childbirth journey has been a relatively recent shift with no special provisions to provide medical, social or psychological support to fathers, even in developed countries [1, 2]. Several qualitative studies of fathers in the perinatal period have identified that fathers wish to be included in perinatal healthcare and engaged by health professionals about their health and wellbeing [3–6]. The demands of reconfiguring roles and identities can be quite confusing and stressful for new fathers [7, 8], however, when complications occur, the stress can be extreme with long-term consequences [6, 9–11].

Traumatic events during pregnancy and childbirth are common in obstetric units [12]. An acute adverse maternal event such as postpartum haemorrhage may occur in 5-15% of births [13]. Additionally, birth-related or neonatal complications may necessitate some form of active resuscitation of the newborn in 16% of cases [14]. Worldwide, more than 3.9 million babies are stillborn each year [15]. Medically indicated terminations of pregnancy are also fairly common occurrences in obstetric units (18.0 per 1000 women in the United Kingdom) [16].

While the psychological consequences of a traumatic perinatal event, such as a fetal loss, are better described for mothers, the impact on fathers is less well understood [17, 18]. One of the reasons for this is the difficulty of recruiting men into studies, particularly after traumatic events [6, 10, 17]. Fathers may display a classical grief response with public grief suppression to conform to the societal expectations of masculinity [6, 19]. There is concern that exposure to traumatic circumstances in pregnancy and childbirth may contribute to post-traumatic stress symptoms (PTSS) in fathers [6, 17, 20–22].

Post-traumatic stress disorder (PTSD) is a persistent syndrome of symptoms relating to traumatic events, which arises 1-30 days after exposure to actual or threatened death or serious injury. It is typically characterised by a range of PTSS, including intrusion of traumatic memories, avoidance, alterations in cognition, mood, arousal and reactivity [23]. Precipitating events for PTSD include direct exposure, witnessing trauma to others, indirect experience by a family member or other close associate and repeated or extreme exposure to aversive details of a traumatic event [23].

Paternal stress reactions have not been studied extensively. Fathers witnessing life-threatening maternal or neonatal events may be left with long-standing PTSS [6, 9, 17, 20–22, 24]. Parental PTSS are associated with exposure to hospital environments (e.g. sights and sounds similar to the neonatal intensive care unit [NICU]), anxiety, depression, paranoid ideation and phobic anxiety [25]. A systematic review highlighted the risk in fathers of developing PTSD after a stillbirth [26]. Furthermore, after a fetal loss, up to 16% of fathers may develop PTSD in the subsequent pregnancy, although these symptoms tend to remit postnatally, after the birth of a live baby [17, 27]. PTSS may be long-standing in up to 8.4% of fathers even up to 18 years after infant loss [28, 29]. As noted by a systematic review, the severity of PTSD is not influenced by the timing of the loss; however, lower gestational age is associated with more severe symptoms [30]. Despite this, support services around the world tend to focus mainly on the mother [31]. The aim of this study was to explore the prevalence of traumatic and depressive symptoms over time in fathers following poor fetal, neonatal and maternal outcomes.

## Methods

### Study design

This is a prospective mixed-methods study of 28 fathers conducted at an outer metropolitan public teaching hospital in Brisbane, Australia delivering approximately 1750 babies each year. Ethics approval was obtained from The Prince Charles Hospital Human Research Ethics Committee (HREC/13/QPCH/188). The qualitative findings have been published previously with the quantitative results using validated screening questionnaires reported here [6].

Participants included fathers whose partners had experienced a pregnancy or childbirth complicated by a significant congenital abnormality or aneuploidy, termination of pregnancy, fetal death in-utero, stillbirth, admission to the NICU or special care nursery or significant maternal morbidity, such as a life-threatening postpartum haemorrhage or an emergency postpartum hysterectomy. These fathers were recruited sequentially via antenatal clinics, attendance at ultrasound appointments or through the postnatal ward between September 2013 and March 2015. Fathers were screened using two scales: the Edinburgh Postnatal Depression Scale (EPDS), to assess symptoms of depression in the perinatal period, and the Impact of Events Scale-Revised (IES-R), to assess subjective distress in response to trauma. Both scales were administered at 2-3 weeks (timepoint 1) and 3-4 months (timepoint 2) after the event. Participants who scored high (≥10 on the EPDS and ≥33 on the IES-R) were offered immediate referral to a mental health practitioner; any participants who experienced distress as part of the interview or questionnaire process were offered referral to a mental health or general practitioner.

### Edinburgh Postnatal Depression Scale

The EPDS is a 10 item self-report questionnaire widely used as a screening tool for perinatal depression in women [32]. The EPDS has also been validated for men in several studies from diverse populations [33–46]. It has one question on self-harm (question 10) and each item has four possible responses from 0 to 3 depending on their severity, with a maximum total score of 30 [32] Three specific items in the EPDS (3, 4 and 5), focus on anxiety in both antepartum and postpartum settings [47, 48]. According to a Swedish validation study, items 3, 4, 5, 7, 8 and 9 reflect unhappiness, which the authors describe as “distress” [39].

The internal consistency of the EPDS in men has been reported to be high (Cronbach’s alpha = 0.81-0.83 and Spearman Brown split half reliability = 0.78) [34, 39, 41]. The sensitivity in identifying postnatal depression ranges from 71% to 100%, with specificity ranging from 75% to 97% [34, 38, 39, 49]. Reported thresholds for depression range from >5 to >12 [34, 35, 49, 50]. We chose a threshold of ≥10 as representing significant symptoms of depression in keeping with most of the literature [34, 37, 38, 41, 51, 52].

### Impact of Events Scale-Revised

The IES-R is the most widely used self-report measure for traumatic stress. It was initially developed by Horowitz et al. [53] using 15 items with two subscales (intrusion and avoidance) and later modified by Weiss with an added subscale of hyperarousal [54]. The revised version comprises 22 items which are divided into 3 sections that are rated from 0-4 on a 5-point Likert scale and measure PTSD symptoms experienced in the past 7 days. The scale takes under ten minutes to administer and does not require any special training as a pre-requisite. The IES-R is usually scored as a total (with a maximum score of 88), although individual subscales can also be reported separately.

The scale has been validated in multiple settings of relevance, such as Vietnam war veterans, in persons after a brain injury, motor vehicle accident survivors, cancer patients and earthquake survivors [55–62]. Additionally, this scale has also been used to examine the impact of preterm birth requiring NICU admission on fathers [10]. The chosen cut-off is ≥33 as it likely signifies PTSS [55].

### Clinical definitions

‘Anticipatory’ events were defined as situations with some warning or time to prepare, such as a diagnosis of a major structural malformation or aneuploidy, which often culminated in a medically advised termination of pregnancy. ‘Sudden’ events were defined as catastrophic events, such as a fetal death, stillbirth, unexpected resuscitation of the newborn or a major postpartum haemorrhage (sometimes requiring a life-saving hysterectomy). Comparisons were made between fathers experiencing anticipatory and sudden events and between fathers who suffered no fetal loss vs. fathers who experienced a fetal loss. The authors made the decision to explore the differences between these subgroups after a preliminary review of the qualitative data [6].

### Statistical methods

Categorical variables were summarised as frequency and percentage and continuous variables as mean and standard deviation (SD) or median and interquartile range (IQR). Pearson’s correlation coefficient (*ρ*) was calculated for EPDS and IES-R comparisons between timepoints 1 and 2. *P*-values less than 0.05 were considered statistically significant. Comparison of anticipatory vs. sudden and fetal loss vs. no fetal loss focus on clinically significant differences in group estimates without statistical tests, due to sample size limitations. Statistical analysis was completed using SPSS Version 25 (IBM Corp, Armonk, NY, USA).

## Results

A total of 32 eligible fathers were invited to participate in the study and 28 accepted. Twenty-seven of 28 participants recruited into the study completed at least one scale. At timepoint 1, 27 fathers completed the EPDS, and 26 fathers completed the IES-R. At timepoint 2, 15 (55.6%) fathers completed both scales. Only a total score for the EPDS response was available for one father at timepoint 1 and one father completed the IES-R at timepoint 2, but not at timepoint 1.

Thirteen fathers had maternal and/or fetal concerns, but took home a live baby, and 14 fathers suffered fetal loss in the form of a medically advised termination of pregnancy (9), fetal death in utero (4) and a stillbirth (1). Most of the participants (85.2%) experienced a fetal complication (Table 1).

**Table 1:** Reasons for inclusion in the study

**Maternal concerns (live baby)**	
Massive postpartum haemorrhage, maternal intensive care admission (2) Fetal structural anomaly, massive postpartum haemorrhage, maternal intensive care admission.	
**Fetal concerns (live baby)**	
Fetal structural anomaly (3), one with special care nursery admission Fetal genetic syndrome Neonatal intensive care admission Intrauterine growth restriction	
**Maternal and fetal concerns (live baby)**	
Preterm delivery and maternal co-morbidities (2), one with NICU admission ^a^Trisomy, preterm delivery ^b^Trisomy, preterm delivery	
**Termination of pregnancy**	
Fetal structural anomaly (4) Trisomy (4) Maternal co-morbidities (1)	
**Fetal death/Stillbirth**	

Fetal death in utero (4) Stillbirth (1)	

^a^ Diagnosed at birth. ^b^Declined termination. *NICU* Neonatal intensive care unit

Table 2 summarises the demographic details of study participants. The mean age of the participants was 33 years (SD 7). Most of the participants were Caucasian (92.6%), employed (76.0%) and had at least one previous child (78.3%) at the time of recruitment into the study.

**Table 2:** Participants characteristics by anticipatory and sudden event types

Characteristic	Overall	Anticipatory	Sudden
	(*n=*27)	(*n=*13)	(*n=*14)
	n (%)	n (%)	n (%)
Age (years, mean (SD))	33 (6.5)	31 (7.3)	35 (5.3)
Ethnicity			
Caucasian	25 (92.6)	12 (92.3)	13 (92.9)
Southeast Asian	2 (7.4)	1 (7.7)	1 (7.1)
Employment status (*n=*25)			
Employed	19 (76.0)	9 (81.8)	10 (71.4)
Unemployed	6 (24.0)	2 (18.2)	4 (28.6)
Nature of complication			
Fetal	23 (85.2)	11 (84.6)	12 (85.7)
Fetal and maternal	1 (3.7)	1 (7.7)	0 (0.0)
Maternal	3 (11.1)	1 (7.7)	2 (14.3)
Number of other children in household (*n=*23)			
None	5 (21.7)	3 (27.3)	2 (16.7)
1	9 (39.1)	6 (54.5)	3 (25.0)
2	5 (21.7)	2 (18.2)	3 (25.0)
3 or more	4 (17.4)	0 (0.0)	4 (33.3)

EPDS and IES-R scores are summarised in Table 3. Participants meeting clinically important cut-offs are described in Table 4. The mean total EPDS score at timepoint 1 was 10.3 (SD 5.7) and 8.0 (SD 6.7) at timepoint 2. The median total IES-R score at timepoint 1 was 30.5 (IQR: 11.0 – 46.3) and 20.0 (IQR: 4.0 – 43.0) at timepoint 2.

**Table 3:** Comparison of EPDS and IES-R scores by anticipatory and sudden event types

Scale and score	Value	Overall	Anticipatory	Sudden
EPDS T1: Total score	n	27	13	14
	mean (SD)	10.3 (5.7)	10.5 (5.2)	10.1 (6.3)
EPDS T2: Total score	n	15	7	8
	mean (SD)	8.0 (6.7)	5.6 (5.5)	10.1 (7.2)
Change in EPDS	n	15	7	8
	median (IQR)	-1.0 (-6.0 - 0.0)	-6.0 (-8.0 - 0.0)	-0.5 (-3.3 - 2.5)
IES-R T1: Intrusion	n	26	13	13
	median (IQR)	1.4 (0.6 - 2.6)	1.9 (0.6 - 2.6)	1.1 (0.6 - 2.4)
IES-R T2: Intrusion	n	15	7	8
	median (IQR)	0.7 (0.3 - 2.4)	0.6 (0.0 - 2.4)	1.5 (0.3 - 2.5)
Change in IES-R Intrusion	n	14	7	7
	median (IQR)	-0.5 (-0.9 - -0.1)	-1.0 (-1.1 - 0.1)	0.0 (-0.4 - 0.1)
IES-R T1: Avoidance	n	26	13	13
	median (IQR)	1.6 (0.4 - 2.3)	1.9 (1.1 - 2.4)	1.1 (0.2 - 1.7)
IES-R T2: Avoidance	n	15	7	8
	median (IQR)	1.3 (0.3 - 1.9)	0.6 (0.4 - 2.4)	1.4 (0.2 - 1.8)
Change in IES-R Avoidance	n	14	7	7
	median (IQR)	-0.1 (-0.7 - 0.2)	-0.5 (-1.8 - 0.1)	0.1 (-0.1 - 0.4)
IES-R T1: Hyperarousal	n	26	13	13
	median (IQR)	1.1 (0.3 - 1.8)	1.1 (0.6 - 1.8)	0.7 (0.2 - 1.8)
IES-R T2: Hyperarousal	n	15	7	8
	median (IQR)	0.4 (0.0 - 1.3)	0.1 (0.0 - 1.3)	0.6 (0.1 - 1.6)
Change in IES-R Hyperarousal	n	14	7	7
	median (IQR)	-0.3 (-1.1 -0.1)	-0.9 (-2.1 - -0.4)	-0.1 (-0.6 - 0.1)
IES-R T1: Total score	n	26	13	13
	median (IQR)	30.5 (11.0 - 46.3)	38.0 (17.0 - 48.5)	17.0 (11.0 - 43.5)
IES-R T2: Total score	n	15	7	8
	median (IQR)	20.0 (4.0 - 43.0)	10.0 (4.0 - 45.0)	24.0 (6.5 - 40.3)
Change in IES-R	n	14	7	7
	median (IQR)	-5.0 (-18.8 - 2.3)	-17.0 (-37.0 - -4.0)	1.0 (-6.0 - 3.0)

**Table 4:** Comparison of clinically important cut-offs for EPDS and IES-R at each timepoint by anticipatory and sudden event types

Scale	Cut-off	Timepoint	n	Overall ***n=***27	Anticipatory ***n=***13	Sudden ***n=***14
				n (%)	n (%)	n (%)
EPDS	≥10	1	27	16 (59.3)	8 (61.5)	8 (57.1)
		2	15^b^	6 (40.0)	2 (28.6)	4 (50.0)
EPDS Q9 (crying)	yes	1	26^a^	18 (69.2)	10 (76.9)	8 (61.5)
		2	15^b^	6 (40.0)	1 (14.3)	5 (62.5)
EPDS Q10 (self-harm)	yes	1	26^a^	4 (15.4)	3 (23.1)	1 (7.7)
		2	15^b^	3 (20.0)	1 (14.3)	2 (25.0)
IES-R	≥33	1	26^a^	12 (46.2)	7 (53.8)	5 (38.5)
		2	15^b^	4 (26.7)	2 (28.6)	2 (25.0)

^a^At timepoint 1, *n=*13 for sudden; ^b^At timepoint 2, for *n=*7 anticipatory and *n=*8 for sudden

Sixteen of 27 fathers (59.3%) at timepoint 1 and 6/15 fathers (40.0%) at timepoint 2 had EPDS scores ≥10; 5 fathers with high EPDS scores (range: 11-23) and 4 fathers with low EPDS scores (range: 4-7) declined to do the scales at the second timepoint. Three fathers with high scores at timepoint 1 scored <10 at timepoint 2. At timepoint 2, 7/15 (46.7%) fathers had no change or an increase in total score; one participant had an EPDS increase of 5 points.

Twelve out of 26 (46.2%) fathers at timepoint 1 and 4/15 (26.7%) fathers at timepoint 2 had IES-R scores ≥33. At timepoint 2, four fathers had persistently high scores: two with extremely high scores (57 and 77). Five fathers with high IES-R scores (range: 38-59) at timepoint 1 did not complete the scale at timepoint 2. Additionally, two fathers had a worsening score, and one stayed at the same high score. No fathers with a score <33 at timepoint 1 displayed trauma-related stress symptoms at timepoint 2.

Similar percentages of fathers in the anticipatory and sudden groups scored ≥10 on the EPDS at timepoint 1 (8/13 [61.5%] vs. 8/14 [57.1%], respectively), but at timepoint 2 more fathers in the sudden group scored ≥10 on the EPDS (2/7 [28.6%] vs. 4/8 [50.0 %], respectively). Although the percentage of fathers with an IES-R score ≥ 33 at timepoint 1 was higher for the ‘anticipatory’ group (7/13 [53.8%] vs. 5/14 [38.0%,], respectively), at timepoint 2 there was little difference between the two groups (2/7 [28.6%] vs. 2/8 [25.0%], respectively). At timepoint 1, 4/26 (15.4%) fathers reported thoughts of self-harm. Whilst only one of these four fathers completed the EPDS at timepoint 2, they continued to report having thoughts of self-harm. Two fathers started having thoughts of self-harm at timepoint 2, with 3/15 (20.0%) fathers exhibiting thoughts of self-harm at this timepoint (Table 4).

The group with the highest scores were participants dealing with fetal loss (Supplementary Tables S1 and S2). Fathers who experienced a fetal loss at timepoint 1 (*n=*14) had a mean EPDS total score of 10.9 (SD 5.3) compared to 9.5 (SD 6.2) for fathers who did not have a fetal loss (*n=*13). Seven fathers with fetal loss had very high IES-R scores (range: 43-62) at timepoint 1. Of the fathers who experienced a fetal loss, 5 declined to do the scales at timepoint 2, although two of these fathers had very high scores on EPDS [17, 23] and three fathers had very high scores on IES-R [44, 46, 58]. In three fathers with a fetal loss, the EPDS and IES-R scores remained persistently elevated suggesting high levels of trauma-based distress (EPDS range at timepoint 1: 10-16; EPDS range at timepoint 2: 10-18; IES-R range at timepoint 1: 43-62; IES-R range at timepoint 2: 43-57), compared to one father who did not have a fetal loss (EPDS scores of 20 and 19 and IES-R scores of 76 and 77 at timepoints 1 and 2, respectively).

None of the fathers who scored low at timepoint 1 (EPDS score <10; IES-R score <33) had high scores at timepoint 2, however, a significant proportion of fathers (40.0% [6/15]) stayed persistently high and some high scores became worse. Additionally, there was a strong positive correlation between the IES-R and EPDS total scores for both timepoints (timepoint 1: *ρ* = 0.787, *p<*0.001; timepoint 2: *ρ* = 0.859, *p<*0.001) (Supplementary Figures 1 and 2).

## Discussion

This study explored the prevalence of symptoms of depression and traumatic stress in fathers in the setting of adverse fetal, neonatal, and maternal outcomes. High scores on screening with EPDS were found in 16/27 (59.3%) fathers at timepoint 1 (2-3 weeks) and 6/15 (40.0%) of fathers at timepoint 2 (3-4 months). Additionally, 6/15 (40.0%) fathers reported crying, and 3/15 (20.0%) reported self-harm thoughts even at 3-4 months following the event. Furthermore, as evident on screening with IES-R, trauma-related stress symptoms were prevalent in 12/26 (46.2%) fathers at 2-3 weeks and 4/15 (26.7%) fathers at 3-4 months. Whilst fathers in both anticipatory and sudden groups experienced these consequences, these feelings were slightly more common in fathers who experienced an anticipatory event at 2-3 weeks. The fathers who suffered a fetal loss had some of the highest scores in the study. Fathers with diverse experiences may need specific assistance at certain times along with extra support. Although these results should be interpreted with caution due to the small number of participants included in our study, they provide insight into fathers at risk of trauma-related stress symptoms.

The impact of childbirth on fathers has been primarily understood from indirect (second-hand) accounts from women regarding their partner's reaction [9, 17, 31]. This study fills a gap in the literature by highlighting the effect of traumatic circumstances during pregnancy and childbirth using self-reported, validated questionnaires. It suggests the need for more widespread screening and assessment of fathers for paternal perinatal depression (PPD), a major depressive disorder in men during pregnancy and following the birth [23]. It also highlights the potential need to assess fathers’ mental health status earlier in pregnancy.

Ours is one of the few studies that provides a longitudinal assessment at two timepoints providing information on paternal response to traumatic circumstances over time. Some of the other studies in the literature have used EPDS to assess fathers at six weeks and six months [43], three days and six weeks [45], two months and six months, [51] and three days, two weeks and six weeks after childbirth [63]. However, none of these studies were conducted in the setting of a traumatic event.

Compared to the published literature, our study suggests a much higher rate of self-reported emotional distress in fathers after a traumatic event (mean score = 10.3 [SD 5.0]). Matthey et al. [34] screened fathers at 6-7 weeks postpartum (using an EPDS cut-off of 5/6 for “distress”) to evaluate the effectiveness of an intervention for postnatal distress. The fathers who were distressed had a higher score (mean = 9.4 [SD 5.0]) when compared to non-distressed fathers (mean score = 4.1 [SD 3.5]) [34]. Another study by Edmondson et al. [37], reported on fathers screened for depression at seven weeks postpartum, after no significant defined event (using an EPDS threshold of >10). Fathers with depression (diagnosed on a structured clinical interview) scored much higher (mean score = 14.8 [SD 3.4]) compared to non-depressed fathers (mean score = 6.6 [SD 4.4]). The results of our study are similar to the study by Matthey et al. [34], however, our results contrast with the Edmondson study [37]. Although our scores are not as high as the fathers in the 'depressed' group in the Edmondson study, our fathers appear to be more distressed than the 'non-depressed' fathers in their study [37]. This may be potentially due to smaller numbers in our study and inherent differences caused by exposure to traumatic circumstances. Future studies with larger sample sizes and extended pre and post follow-up periods are needed to evaluate the long-term effect of exposure to traumatic events on fathers.

The rates of thoughts of self-harm identified in the literature are low and include figures of 4% in routine postpartum settings [34] and 3% in fathers scoring ≥12 on the EPDS [39]. Although the numbers in our study are small, it shows a much higher prevalence of thoughts of self-harm (20.0%) than those reported in the literature [34, 39, 64]. However, our rates are similar to an Egyptian study that reported suicidal ideation in 18.5% of antenatally depressed fathers using an EPDS cut-off of ≥10, with the background prevalence of symptoms suggestive of antenatal depression relatively high at 31.8% [52].

The findings from our study highlight the high frequency of depression and traumatic symptoms that can persist for months after the event. Given these findings, it is concerning that fathers are not routinely screened for mental health concerns before or during pregnancy or in the postpartum period. The fathers in our study who had screening results consistent with significant mental health challenges had ongoing responsibility to care for their partner, baby and other children depending on the situation. Some of these men may also father another child particularly after a stillbirth and termination of pregnancy. This has important implications as fathers have a profound impact on their offspring's future [65, 66]. Additionally, PTSD and depression in parents significantly affect infant development and behaviour [25]. Therefore, the findings of our study highlight an urgent need to provide support and intervention for fathers who have experienced adverse fetal, maternal, and neonatal outcomes.

Almost one-third of fathers (26.7%) in our study had IES-R scores of ≥33 at 3-4 months, suggesting the presence of PTSS. This exemplifies the need for appropriate clinical assessment for PTSD. This is an important area for further study as the risk of suicide is significantly increased in individuals with PTSD [67]. The total IES-R scores in our study were much higher when compared to a study of fathers of preterm infants (gestational age <37 weeks) admitted to the NICU, indicating elevated general stress levels (i.e., an IES-R total median score = 20.0 [IQR 4.0 – 43.0] at timepoint 2 compared to IES-R total mean score = 1.6 [SD = 1.3], respectively) [10]. Compared to the Ionio et al. study [10], we also found higher subscale scores in fathers who experienced a sudden event (intrusion median 1.5 [IQR 0.3 - 2.5] and hyperarousal median 0.6 (IQR 0.1- 1.6) versus intrusion mean = 0.6 [SD = 0.6] and hyperarousal mean = 0.5 [SD = 0.8], respectively) [10].

A study of fathers whose infants were admitted to the NICU, highlighted that within 2-4 weeks of the event, fathers appeared to cope better by delaying their own emotional response [25]. However, when examined four months after the event, 33% of fathers met the diagnostic criteria for PTSD, suggesting a delayed onset and heightened risk. When comparing our results with the study by Shaw et al., our findings are contrasting, as the prevalence of PTSD in fathers decreased by half between 2 and 4 weeks of the acute stress (i.e., 67% to 33%). However, the study by Shaw et al. highlights the potentially increased risk for subsequent development of PTSD in fathers, despite exhibiting minimal symptoms of acute stress disorder 2-4 weeks after a precipitating event [25]. Given this variability in outcomes related to the timing of PTSD assessment, future research needs to explore the time course associated with perinatal PTSS.

### Limitations

There are several limitations in this study that deserve mention. The modest sample size and high attrition rate reflect the difficulty of recruiting and retaining fathers in a longitudinal study design of this nature and may impact our point estimates of traumatic symptoms. We believe our estimates at timepoint 2 may be conservative as the fathers who declined scales at timepoint 2 had high rates of initial distress and may have decided not to participate due to ongoing distress. The study was also underpowered to examine the differences between subgroups. Exploring the differences between sudden and anticipatory events deserves attention in further studies. We did not have a control group of fathers or pre-event details on the prevalence of mental health symptoms; hence the authors relied on the published literature to compare the findings of this study. Additionally, we did not screen for symptoms in the men’s partners, which may have impacted on the mental health of fathers. This was a single-centre study with most participants of Caucasian background, and this limits generalisability to culturally diverse settings. Furthermore, heterogeneity across the traumatic experiences of fathers and the wide range of cut-offs used for the EPDS and IES-R in the literature makes comparison difficult.

## Conclusion

This study provides evidence that fathers who experienced a traumatic pregnancy or perinatal event may have high levels of distress, including thoughts of self-harm, in the observed 3-4 months after the event. The numbers from this study are likely to underestimate the magnitude of this problem. Further research should be directed to confirm the findings from this study in a larger sample of geographically, culturally, and linguistically diverse populations. The value of screening for mental health conditions in men during pregnancy and after birth needs to be examined. Interventional studies to explore how to improve paternal outcomes are urgently required. Given screening questionnaires suggest a high burden of paternal mental health issues after traumatic obstetric events, it is essential to evaluate the long-term consequences of this on men, their partners, and their children.

## Supplementary Information

**Additional file 1: Table S1.** Comparison of EPDS and IES-R scores by fetal outcome.

**Additional file 2: Table S2.** Comparison of clinically important cut-offs for EPDS and IES-R and EPDS question 9 and 10 at each timepoint by fetal outcome.

**Additional file 3: Fig. 1.** Correlation between IES and EPDS total scores for time point 1.

**Additional file 4: Fig. 2.** Correlation between IES-R and EPDS total scores for time point 2.

## Data Availability

The datasets generated and/or analysed during the current study are not publicly available due to patient confidentiality and the sensitive nature of the information, but are available from the corresponding author on reasonable request.

## Abbreviations

EPDS: Edinburgh Postnatal Depression Scale
IES-R: Impact of Events Scale-Revised
NICU: Neonatal Intensive Care Unit
PPD: Paternal perinatal depression
PTSD: Post-traumatic stress disorder
PTSS: Post-traumatic stress symptoms
