# Correction: Dads in Distress: symptoms of depression and traumatic stress in fathers following poor fetal, neonatal, and maternal outcomes

**DOI:** 10.1186/s12884-023-05395-x

**Published:** 2023-01-24

**Authors:** A. Kothari, G. Bruxner, J. M. Dulhunty, E. Ballard, L. Callaway

**Affiliations:** 1 Redcliffe Hospital, Anzac Avenue, Redcliffe, Queensland 4020 Australia; 2 The University of Queensland, Brisbane, Queensland Australia; 3 QIMR Berghofer Medical Research Institute, Brisbane, Queensland Australia; 4 The Royal Brisbane and Women’s Hospital, Brisbane, Queensland Australia

**Correction: BMC Pregnancy Childbirth 22, 956 (2022)**

**https://doi.org/10.1186/s12884-022-05288-5**

Following publication of the original article [1], the editor reported that insertion of "male" in the Abstract appears to be an error.

The sentence currently reads:

**Methods:** A prospective mixed-methods study was conducted at an outer metropolitan public teaching hospital in Brisbane, Australia, with quantitative results presented here. Subjects included 28 fathers whose **male** partners had experienced pregnancy or childbirth complicated by a significant congenital abnormality or aneuploidy, termination of pregnancy, fetal death in-utero, stillbirth, admission to the neonatal intensive care unit or special care nursery or significant maternal morbidity, such as a postpartum haemorrhage or an emergency postpartum hysterectomy.

The sentence should read:

**Methods:** A prospective mixed-methods study was conducted at an outer metropolitan public teaching hospital in Brisbane, Australia, with quantitative results presented here. Subjects included 28 fathers whose partners had experienced pregnancy or childbirth complicated by a significant congenital abnormality or aneuploidy, termination of pregnancy, fetal death in-utero, stillbirth, admission to the neonatal intensive care unit or special care nursery or significant maternal morbidity, such as a postpartum haemorrhage or an emergency postpartum hysterectomy.
