# Supplementary material for: Dads in Distress: symptoms of depression and traumatic stress in fathers following poor fetal, neonatal, and maternal outcomes
Source: BMC Pregnancy Childbirth. 2022 Dec 22;22:956. doi: 10.1186/s12884-022-05288-5 (PMC9773585; doi:10.1186/s12884-022-05288-5)
Supplement: Supplementary file 1 — Additional file 1: Table S1. Comparison of EPDS and IES-R scores by fetal outcome. [file 12884_2022_5288_MOESM1_ESM.docx]

Table S1: Comparison of EPDS and IES-R scores by fetal outcome

| **Scale and score** | **Value** | **No fetal loss** | **Fetal loss** |
| --- | --- | --- | --- |
| EPDS Scale 1: Total score | n | 13 | 14 |
|  | mean (SD) | 9.5 (6.2) | 10.9 (5.3) |
| EPDS Scale 2: Total score | n | 6 | 9 |
|  | mean (SD) | 8.7 (8.0) | 7.6 (6.1) |
| Change in EPDS | n | 6 | 9 |
|  | median (IQR) | 0.0 (-4.8 - 2.0) | -4.0 (-6.0 - 0.0) |
| IES-R Scale 1: Intrusion | n | 13 | 13 |
|  | median (IQR) | 1.1 (0.4 - 2.1) | 1.9 (0.9- 2.8) |
| IES-R Scale 2: Intrusion | n | 6 | 9 |
|  | median (IQR) | 0.9 (0.0-2.0) | 0.7 (0.4- 2.5) |
| Change in IES-R Intrusion | n | 6 | 8 |
|  | median (IQR) | -0.4 (-1.1- -0.1) | 0.6 (-1.0 - -0.1) |
| IES-R Scale 1: Avoidance | n | 13 | 13 |
|  | median (IQR) | 1.4 (0.2 - 1.9) | 1.8 (1.0- 2.4) |
| IES-R Scale 2: Avoidance | n | 6 | 9 |
|  | median (IQR) | 0.3 (0.2 - 2.2) | 1.6 (0.6- 2.1) |
| Change in IES-R Avoidance | n | 6 | 8 |
|  | median (IQR) | -0.0 (-0.7 - 0.3) | -0.1 (-1.1- 0.1) |
| IES-R Scale 1: Hyperarousal | n | 13 | 13 |
|  | median (IQR) | 0.7 (0.1 – 1.6) | 1.3 (0.6 – 1.9) |
| IES-R Scale 2: Hyperarousal | n | 6 | 9 |
|  | median (IQR) | 0.5 (0.1- 1.6) | 0.4 (0.0- 1.5) |
| Change in IES-R Hyperarousal | n | 6 | 8 |
|  | median (IQR) | 0.5 (-0.6 - 0.3) | 0.9 (-1.1- 0.1) |
| IES-R Scale 1: Total score | n | 13 | 13 |
|  | median (IQR) | 24.0 (6.5 - 41.0) | 43.0 (16.0 – 48.0) |
| IES-R Scale 2: Total score | n | 6 | 9 |
|  | median (IQR) | 10.5 (3.8 - 43.3) | 20.0 (8.5- 44.0) |
| Change in IES-R | n | 6 | 8 |
|  | median (IQR) | -1.5 (-15.5 - 3.0) | -12 (-22.3- 2.3) |
