# Supplementary material for: Dads in Distress: symptoms of depression and traumatic stress in fathers following poor fetal, neonatal, and maternal outcomes
Source: BMC Pregnancy Childbirth. 2022 Dec 22;22:956. doi: 10.1186/s12884-022-05288-5 (PMC9773585; doi:10.1186/s12884-022-05288-5)
Supplement: Supplementary file 2 — Additional file 2: Table S2. Comparison of clinically important cut-offs for EPDS and IES-R and EPDS question 9 and 10 at each timepoint by fetal outcome. [file 12884_2022_5288_MOESM2_ESM.docx]

Table S2: Comparison of clinically important cut-offs for EPDS and IES-R and EPDS question 9 and 10 at each timepoint by fetal outcome

| **Scale** | **Cut-off** | **Time point** | **n** | **No fetal loss** | **Fetal loss** |
| --- | --- | --- | --- | --- | --- |
|  |  |  |  | n=13 (%) | n=14 (%) |
| EPDS | ≥10 | 1 | 27 | 8 (61.5) | 6 (42.9) |
|  |  | 2 | 15^~^ | 3 (50.0) | 3 (33.3) |
| EPDS Q9 (crying) | yes | 1 | 26^ | 7 (53.8) | 11 (84.6) |
|  |  | 2 | 15^~^ | 3 (50.0) | 3 (33.0) |
| EPDS Q10 (self-harm) | yes | 1 | 26^ | 1 (7.7) | 3 (23.1) |
|  |  | 2 | 15^~^ | 1 (16.7) | 2 (22.2) |
| IES-R | ≥33 | 1 | 26^ | 5 (38.5) | 7 (53.8) |
|  |  | 2 | 15^~^ | 1 (16.7) | 3 (33.3) |

^ At timepoint 1 for sudden n=13; ^~^ At time point 2 for no fetal loss n=6 and for fetal loss n=9
