# Supplementary figures and images for: Dads in Distress: symptoms of depression and traumatic stress in fathers following poor fetal, neonatal, and maternal outcomes
Source: BMC Pregnancy Childbirth. 2022 Dec 22;22:956. doi: 10.1186/s12884-022-05288-5 (PMC9773585; doi:10.1186/s12884-022-05288-5)

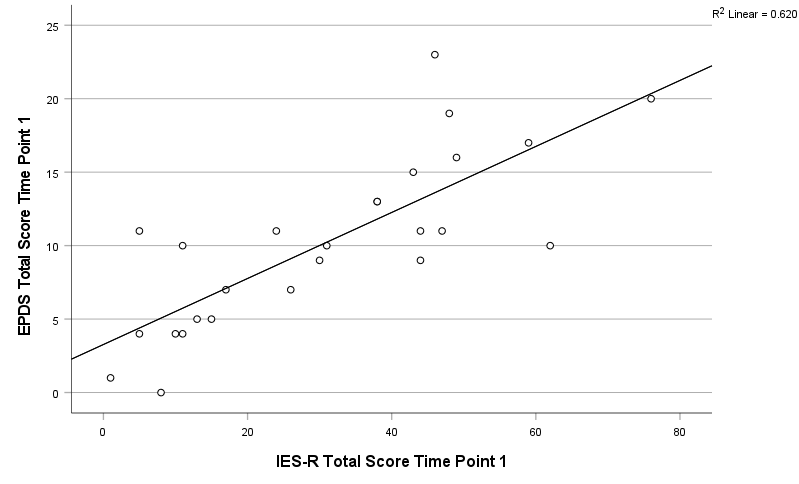


Figure 1: Correlation between IES and EPDS total scores for time point 1.

Supplement: Supplementary file 3 — Additional file 3: Fig. 1. Correlation between IES and EPDS total scores for time point 1. [file 12884_2022_5288_MOESM3_ESM.docx]

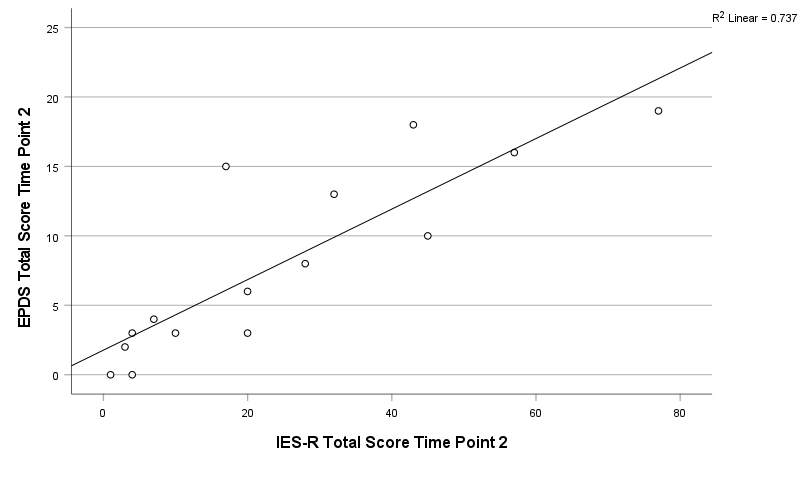


Figure 2: Correlation between IES-R and EPDS total scores for time point 2.

Supplement: Supplementary file 4 — Additional file 4: Fig. 2. Correlation between IES-R and EPDS total scores for time point 2. [file 12884_2022_5288_MOESM4_ESM.docx]
